# Influence of Food Processing on Blood Lipids in Children

**DOI:** 10.3390/nu8020097

**Published:** 2016-02-18

**Authors:** Marcia R. Vitolo, Fernanda Rauber

**Affiliations:** Federal University of Health Sciences of Porto Alegre (UFCSPA), Rua Sarmento Leite 245, Porto Alegre 90050-170, Brazil; rauber.fernanda@gmail.com

With reference to a recent study published in this journal “*Processed Food Contributions to Energy and Nutrient Intake Differ among US Children by Race/Ethnicity*”, by Eicher-Miller *et al*. [1], we would like to make some comments, as our study [2] was mentioned and there are some misunderstandings regarding our conclusions. Our longitudinal study investigated the effect of processed and ultra-processed food intake between 4 and 8 years old children on blood lipid profile. We used NOVA—a food classification system that categorizes foods according to the extent and purpose of food processing [3]—which “*is now recognized as a valid tool for nutrition and public health research, policy and action, in reports from the Food and Agriculture Organization of the United Nations and the Pan American Health Organization”* [4]. Our study provided evidence that ultra-processed food expressed as a percentage of total energy intake was associated with worse lipid profiles in children [2]. In the study by Eicher-Miller and colleagues, food intake was assessed using a different food classification proposed by the International Food Information Council Foundation [5]. This system aims to provide information about modern food production, food processing and processed foods to guide consumers and clients, rather than assess the impact of these foods on health. Based on their findings that “minimally processed foods” provided high quantities of cholesterol (according to their methodology) the authors suggested a possible bias in our study since we did not consider the energy provided by minimally processed foods, only from processed and ultra-processed foods. Therefore, according to them, we have lost a potential strong association between “minimally processed foods” and blood lipid concentrations. First of all, the main question of our research was to verify the effect of processed food consumption on children’s lipid profile, adjusting for relevant variables. Second, the author’s interpretation of our study was superficial because our analysis was done using the percentage of total energy from processed and ultra-processed foods and not simply energy. This detail makes a huge difference, since children consuming high percentages of energy provided by processed and ultra-processed food will have a low intake of energy provided from minimally processed foods, and *vice versa*. Third, the Eicher-Miller conclusions must be reviewed going back to their classification of food groups. For instance, in their study the cholesterol content was shown separately in three food categories (mixtures of combined ingredients, ready-to-eat processed foods, prepared foods/meals, and foods from restaurants/cafeterias) that are all ultra-processed foods according to NOVA classification. Taking their Table 1 as an example and summing the cholesterol content of all ultra-processed foods together will result in 64.4 mg of cholesterol, while minimally processed foods contributed 29.2 mg of cholesterol. The same fact can be observed in Tables 2 and 3 (63.5 *vs.* 30.9 and 56.7 *vs.* 40.8, respectively). Fourth, our results show that we did not observe any association between the “processed food category” and blood lipids, which suggests that among children, in our study only ultra-processed foods were associated with cardiovascular risk factors. Finally, as they stated about our results *“Energy of minimally processed foods was not included and may represent a potentially stronger association with blood lipid”*, we would like to add that, so far, there is not enough evidence of a relation between diet cholesterol and blood lipids as concluded by a recent systematic review with adults [6]: “*the effect of dietary cholesterol on incident CAD and serum cholesterol outcomes remains unclear*”.
